# Wearable 3D-Printed Microneedle Sensor for Intradermal Temperature Monitoring

**DOI:** 10.1021/acssensors.4c03681

**Published:** 2025-04-15

**Authors:** Qikun Wei, Daniel Rojas, Qianyu Wang, Ruben Zapata-Pérez, Xing Xuan, Águeda Molinero-Fernández, Gastón A. Crespo, María Cuartero

**Affiliations:** † Department of Chemistry, 7655 KTH Royal Institute of Technology, Teknikringen 30, Stockholm SE-114 28, Sweden; ‡ 16728 UCAM-SENS, Universidad Católica San Antonio de Murcia, UCAM HiTech, Avda. Andres Hernandez Ros 1, Murcia 30107, Spain; § Group of Metabolism and Genetic Regulation of Disease, Universidad Católica San Antonio de Murcia, UCAM HiTech, Avda. Andres Hernandez Ros 1, Murcia 30107, Spain

**Keywords:** microneedle, interstitial fluid, temperature monitoring, 3D printing, conducting polymers

## Abstract

Accurate temperature monitoring plays a crucial role in understanding the physiological status of patients and the early diagnosis of diseases commonly associated with local and global infections. Intradermal temperature measurement is, in principle, more precise than skin surface detection, as it prevents interference from environmental temperature changes and skin secretions. However, to date, precise and reliable intradermal temperature monitoring in a real-time and continuous manner remains a challenge. We propose herein high-resolution 3D printing to fabricate a mechanically robust and biocompatible hollow microneedle, filled with a temperature-responsive conducting polymer (poly­(3,4-ethylenedioxythiophene): polystyrenesulfonate, PEDOT:PSS) to develop a microneedle temperature sensor (T-MN). The significance is 2-fold: rational design of robust MNs with high resolution in the micrometer domain and the implementation of a conducting polymer in a MN format for temperature sensing. The analytical performance of the developed T-MN is in vitro evaluated under mimicked intradermal conditions, demonstrating good sensitivity (−0.74%° C^–1^), resolution (0.2 °C), repeatability (RSD = 2%), reproducibility (RSD = 2%), reversibility, and medium-term stability. On-body temperature monitoring is performed on six euthanized rats for 80 min. The results presented good agreement with those obtained using a commercial optical temperature probe, which was intradermally inserted into the rat skin. The reliability of utilizing the T-MN for precise and continuous intradermal temperature monitoring was successfully demonstrated, noting its potential use for patient monitoring in the near future but also temperature compensation for MN (bio)­sensors that may need it.

## Graphical abstract

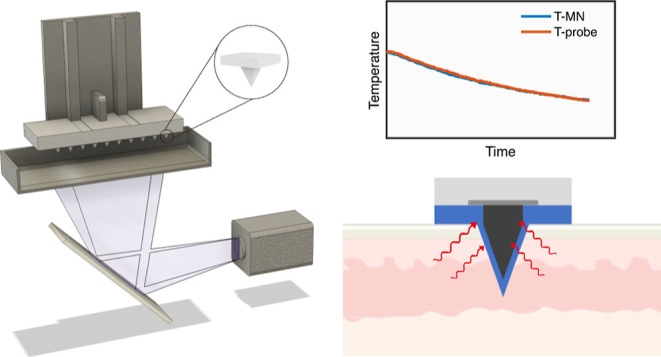

Wearable devices caught remarkable attention in the last five years because of their capability to offer continuous and real-time monitoring of physiological metrics and biomarkers^1^ in several biological fluids such as sweat,^2^ saliva,^3^ urine,^4^ and dermal interstitial fluid (ISF).^5^ Extensive research has been conducted, developing different techniques for sampling and analysis, which is particularly challenging for the ISF, since this is not a peripheral fluid. Notably, the importance of analyzing ISF relies on its similarities with blood in terms of physiological information (e.g., biomarkers’ concentration and evolution) while being more accessible.^6^ As a result, ISF is believed to hold significant potential as an alternative to certain well-established gold-standard blood analyses.

The designs of wearable devices conceived for ISF analysis primarily employ microneedles (MN) as the sensor platform, selected because of their accessibility to dermal ISF, minimal invasiveness, and feasibility for miniaturization and integration of the sensing element.^6,7^ In particular, MN-based sensors with electrochemical readout and targeting diverse biomarkers, such as glucose,^8^ lactate,^9^ glycine,^10^ pH,^11^ potassium,^12^ carbon dioxide,^13^ multiple ions,^14^ etc., have been successfully developed. In essence, these sensors can be classified into two main categories, namely, enzyme-based amperometric MN-(bio)­sensors and potentiometric MN-(bio)­sensors. Importantly, temperature has a significant influence on both signal readouts (current or potential) and, consequently, on the obtained biomarker concentration/activity. Some cases in which the temperature effect tends to be overlooked are (i) the enzyme activity in amperometric MN sensors (linked to conformational changes of enzyme proteins, deactivation); (ii) the slope in the calibration graph of potentiometric MN sensors (in relation to the Nernst equation); and (iii) considering gaseous biomarkers like carbon dioxide, oxygen, and nitric oxide, their solubilities and diffusion coefficients in aqueous biofluids are temperature-dependent.^15,16^ Following this, a few strategies that have already been proposed for temperature correction in MN-based measurements are discussed.

Abbott incorporated an external thermistor module into their continuous ISF glucose monitoring patch (FreeStyle Libre).^17^ However, the readout only reflects the temperature in the proximity of the skin surface rather than in the ISF, where in fact the glucose measurement takes place. It is important to note that the temperature difference between the skin surface and 1 mm depth inside the skin is in the range from 0.6 to 2 °C, depending on environmental temperature, body site of measurement, and physiological conditions.^18,19^ The skin surface temperature measurements are highly susceptible to external environmental factors, including ambient temperature fluctuations and sweat production, which can introduce inconsistencies in their precise measurement aiming at either clinical meanings or the correction of other (bio)­sensor responses. In contrast, intradermal temperature measurement provides a more stable and physiologically relevant parameter within a skin microenvironment in relation to the ISF.^20^ Kim et al.^21^ developed a temperature compensation algorithm for MN glucose electrodes, assuming that the temperature at the measurement site is the same as that at the tip end of the MN glucose sensor. Also, the direction of an implantable temperature sensor placed inside the dermal layer was reported. While promising, the applications are limited by the invasiveness and required surgery.^22^

A simple approach to implementing temperature correction in MN biosensors is to use a MN-based temperature sensor, finding only a few and rather incomplete examples in the literature. He and co-workers^20^ proposed a colorimetric temperature-responsive powder to produce patterned MNs from a polydimethylsiloxane/poly­(methyl methacrylate) (PDMS/PMMA) mold. The temperature measurement was semiquantitative, impairing the necessary accuracy and resolution for applications in temperature compensation for MN biosensors. Sun et al.^23^ presented a laser-cutting MN electrode array based on an Au/Ti thermocouple for both temperature monitoring and biosignal analysis on humans. Unfortunately, there is a lack of systematic characterization of the analytical performance and no validation of any such method at all. Moreover, compared to molding and laser-cutting techniques, 3D printing offers significant advantages for MN fabrication, including high customization of the design, a wide selection of printable materials, cost-efficiency in large-volume production, and high-resolution printing.^24–26^ Indeed, in the last years, there has been increasing interest in the use of this technology for the fabrication of 3D-printed MNs, additionally providing possibilities for biocompatible and mechanical robustness to ensure their safety and reliability when used as (bio)­sensors in clinical settings. To date, there is no report of a 3D-printed MN-based temperature sensor, to the best of our knowledge.

Beyond the described correction, a temperature MN will add significant value from a clinical perspective, both as an independent tool and when combined with other sensors. Temperature is related to the thermal regulation of patients, enabling a deeper understanding of localized inflammatory responses, infections, or ischemic conditions.^27^ Of importance is also its evolution; for example, the temperature monitoring of patients under general anesthesia is known to help prevent hypothermia. Another usage is foreseen in critically ill patients undergoing therapeutic hypothermia for neurological injuries.^28^ Effectively, when integrated with chemical sensors, a temperature MN sensor can serve as a multidimensional diagnostic tool, detecting both temperature changes and key biomarkers like pH, glucose, or lactate levels, which can be used for assessing wound healing or metabolic abnormalities. This could aid in monitoring diabetic ulcers, detecting sepsis at an early stage, or assessing vascular health in peripheral tissues.^29–31^ Furthermore, temperature MNs can advance personalized medicine by facilitating real-time monitoring of individual thermal and chemical profiles, potentially enabling more precise and targeted therapeutic interventions.

Here, we present a MN-based temperature sensor (T-MN) consisting of a 3D-printed MN. High-resolution 3D printing is used to fabricate biocompatible and mechanically robust MNs that are later modified to provide temperature sensing capabilities at the intradermal level. The thermal-responsive conducting polymer poly­(3,4-ethylenedioxythiophene): polystyrenesulfonate (PEDOT:PSS) was selected as the composite to be incorporated in the T-MN. Specifically, the temperature measurement relies on the linear correlation between the electrical resistance of the PEDOT:PSS and the temperature. To mitigate the impact from environmental temperature variations, a thermal insulating layer was additionally incorporated in the upper part of the MN device, ensuring enhanced reliability and accuracy. A systematic evaluation of T-MN is performed to demonstrate its analytical features. Moreover, cytotoxicity tests confirmed the biocompatibility of the T-MN, showcasing its promising potential for in vivo applications in humans. The validation of the T-MN measurements was performed both in vitro and *on body* levels, this latter based on rats’ assays, confirming its ability to provide precise temperature measurements and enable real-time, continuous intradermal temperature. Overall, the findings presented herein pave the way for reliable temperature compensation in MN biosensors while providing valuable physiological information about the state of the traced subject.

## Experimental Section

### Fabrication of the Planar Temperature Sensor Patch (T-Patch)

A planar T-patch was first developed to demonstrate the concept of temperature monitoring based on PEDOT:PSS. The T-patch consisted of a three-layer structure: a substrate layer, a sensing layer, and an electrical insulating layer. Polyester sheets (Mylar A, 0.1 mm, RS Pro) were cut into the desired shape by using a Silhouette Cutter (Silhouette Cameo 3) and used to form each layer. The layers were assembled by using double-sided adhesive tape (300 LSE, 3M). The bottom polyester layer (i.e., the sensor substrate) was designed with dimensions of 2 × 3 cm. The middle layer was cut to accommodate a 6 × 6 mm PEDOT:PSS in the center and two smaller copper sheets (6 × 11 mm) on both sides to establish electrical connections. The top polyester layer served as the electrical insulating film, with the same dimensions as the substrate bottom layer (2 cm × 3 cm).

The T-patch was prepared as follows. First, the substrate and middle layers were assembled using double-sided adhesive tape. Then, two copper sheets were placed in the left and right grooves. Next, 20 μL of the PEDOT:PSS solution was drop-cast on the center space between the copper sheets and polyester film. A PEDOT:PSS film was formed after drying in an oven at 100 °C for 1 h. For ease of connecting the T-patch to a multimeter, two wires were attached to the copper sheets. Finally, an electrically insulating polyester layer was applied on top to complete the assembly. Furthermore, to prevent potential water penetration, the edges of the assembled patch were sealed with hot-melt glue. Figure S1 in the Supporting Information shows the summary scheme for the T-patch preparation.

### Fabrication of T-MN

The hollow MN for holding the temperature-sensing components was designed in Autodesk Fusion 360 and printed using a digital light processing 3D printer (Profluidics 285D, CADworks3D). The printing parameters followed the recommended manufacturing settings for the Clear microfluidic resin (acrylate-based resin, Clear microfluidic resin V7.0a, CADworks3D) by using a layer height of 30 μm. Different geometrical shapes were tested, namely, conical-shaped and pyramidal-shaped with different dimensional parameters (height: 1000 μm, basal diameter/length 200, 400, 600, 800, or 1000 μm). The pyramidal-shaped hollow MNs with a basal length of 1000 μm and a height of 1000 μm presented the best robustness toward skin insertion but also the highest success rate (90%) during manufacturing. The optimized design of the polymeric hollow MN is depicted in Figure 1a together with a real image.

**Figure 1:**
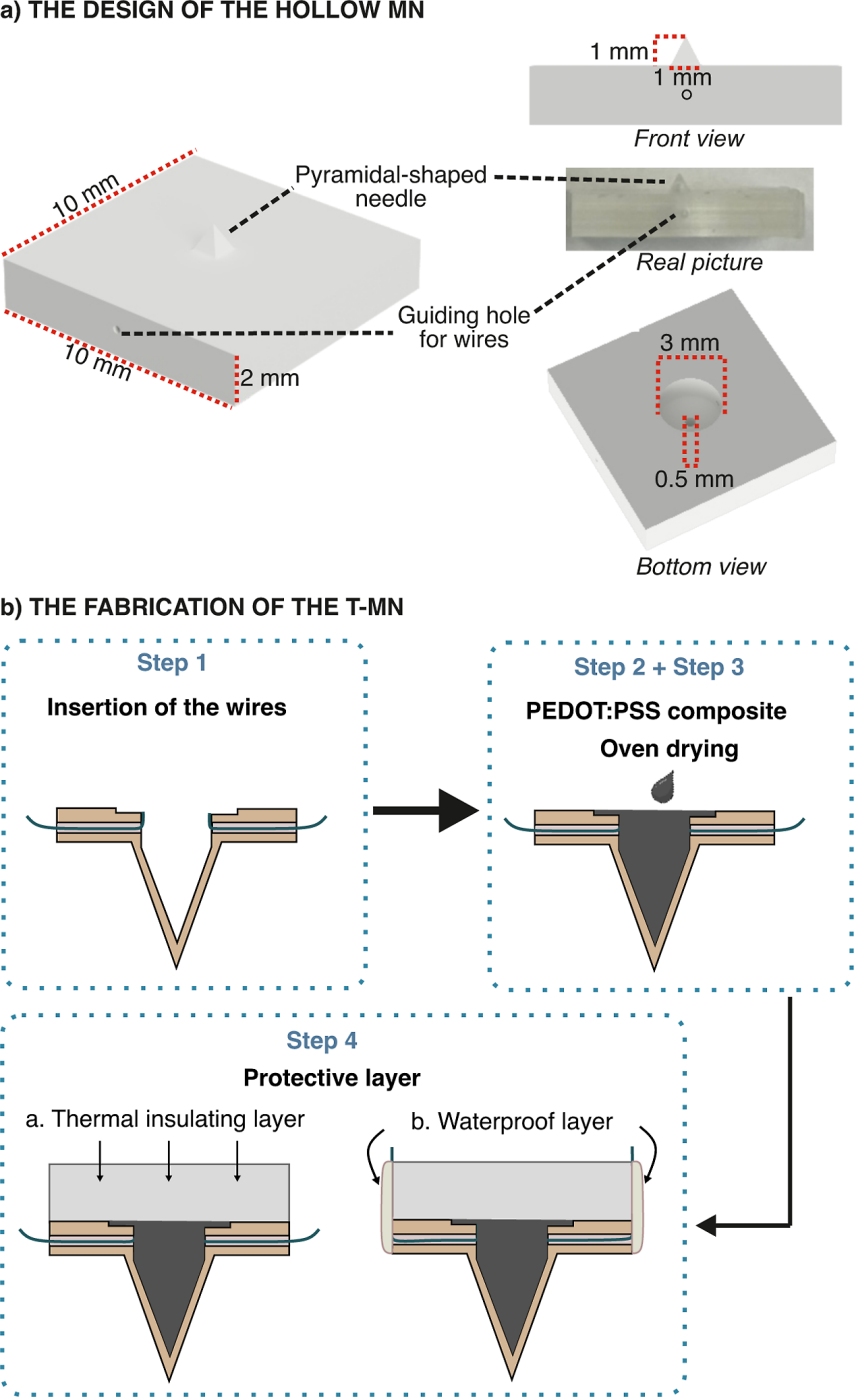
(a) Left: design of the hollow MN performed in Autodesk Fusion 360. Right: front and bottom views with labeled dimensional parameters together with a real image of the 3D-printed hollow MN. (b) Schematic illustration of the process for the preparation of the T-MN.

The steps toward the preparation of the T-MN from the printed hollow MN are illustrated in Figure 1b. First, two stainless steel wires (SS wire, 75 μm) were integrated into the polymeric hollow MN through the wire guiding channels, being securely fixed with super glue (step 1). Then, 15 μL of PEDOT:PSS solution (see the Supporting Information for the composition) was filled into the inner lumen of the hollow MN (step 2) and allowed to dry in the oven at 100 °C for 1 h (step 3). A thermal insulating tape was then cut into a suitable size and attached to the upper part of the MN (step 4a). To prevent the wires from directly contacting water, a layer of electrical insulating hotmelt glue was applied to the lateral sides of the substrate using a hot-melt glue (step 4b). This protective film prevents potential short-circuiting of T-MN during measurements in water.

## Results and Discussion

### Mechanism for Temperature Measurements

PEDOT:PSS was selected as the sensing element in the developed temperature sensors because of its recognized thermal-electrical properties, thermal stability, and facile processability.^32^ PEDOT:PSS is known to exhibit a core–shell structure, where the positively charged chains of PEDOT are surrounded by negatively charged hydrophilic PSS chains. The transport of charge carriers in PEDOT:PSS occurs mainly through the transporting of polarons in the PEDOT chains and the hopping of polarons between the PEDOT segments.^33^ Under thermal stimulus, the transport of charge carriers in PEDOT:PSS is promoted and more charge carriers are activated, synergistically leading to an enhancement of the electrical conductivity. This mechanism is illustrated in Figure 2.

**Figure 2:**
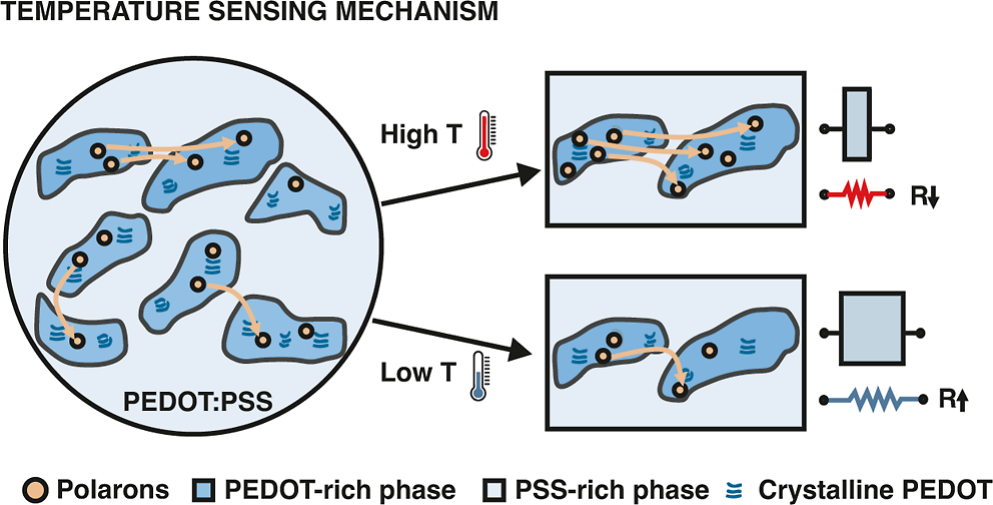
Illustration of the mechanism for charge carrier transport in the PEDOT:PSS film and the corresponding temperature influence.

The integration of PEDOT:PSS into different platforms can be performed by an easy drop-casting process, which can be advantageously automatized via nanodispensing or even direct 3D printing in a more advanced approach.^34^ Furthermore, the PEDOT:PSS technology herein developed is versatile and can be incorporated into any electrode (sensor) format. In contrast, other materials such as metals, metal oxides, and semiconductors will require complicated manufacturing procedures to be implemented in the MN format. In addition, the thermal-electrical properties of PEDOT:PSS make it an adequate material for temperature sensing. It is here anticipated that the sensitivity of the developed T-MN is comparable to and even higher than some resistance temperature detectors or thermistors (see Table S1).

To enhance the sensitivity and stability of the PEDOT:PSS-based temperature sensor, we have adopted the strategy of adding a cross-linker [(3-glycidyloxypropyl) trimethoxysilane (GOPS)] and a nonionic surfactant (Triton X-100) in the preparation of the conducting polymer composite solution. Specifically, GOPS can form chemical linkages between the PSS chains and the underlying polyester/polyacrylate substrate, thus preventing the delamination of the PEDOT:PSS film from the MN, hence increasing the stability during its long-term usage. Cross-linking reactions can also occur between methoxysilane groups on GOPS molecules and sulfonate groups in PSS chains, thus restricting the transport of charge carriers in the PEDOT:PSS grains and thus facilitating the improvement of the sensitivity in PEDOT:PSS-based temperature sensors.^35^ Effectively, Triton X-100 was confirmed to improve the electrical conductivity of PEDOT:PSS by enhancing the crystalline packing of PEDOT segments.^36^ As a surfactant, Triton X-100 also decreases the surface tension of the PEDOT:PSS solution, which is expected to promote the formation of homogeneous films on the polyester/polyacrylate substrate.

### Optimization of the PEDOT:PSS Solution

The temperature sensing performance of the PEDOT:PSS was first investigated in a planar sensor format, which is to be later translated into the MN configuration. Calibrations were conducted in a water bath (Figure S2), utilizing a magnetic hot plate for temperature control and a multimeter for recording the resistance of the composite in the T-patch at different temperatures. The provided signal was treated as *R*/*R*_0_, dividing the resistance at the tested temperature by the resistance at the first set temperature (25 °C). Various compositions of the PEDOT:PSS solutions used for its deposition in the T-patch were examined, comparing the manifested sensitivity toward temperature changes. In essence, this can be tuned by adjusting the weight ratio of GOPS to PEDOT:PSS (from 5:1 to 13:1, C1–C5 in Table S2) in the mixture solution, while keeping the amount of Triton X-100 and PEDOT:PSS constant.

Notably, the sensitivity was quantified as the temperature coefficient of resistance (TCR), using eq 1

1 $$TCR=\frac{R-R_{0}}{R_{0}}\times \frac{1}{T-T_{0}}\times 100\%$$

where *R* is the resistance of the T-patch at the measured temperature, *R*_0_ is the resistance at the initial set temperature (*T*_0_), and *T* represents the measured temperature.

The calibration curves of the T-patches prepared with different compositions, together with the revealed TCR values, are presented in Figure 3. In all the cases, linearity was found in the temperature range from 25 to 50 °C. An increase in the amount of GOPS in the composite translated into an increase in the sensitivity (from C1 to C4), reaching a plateau from the GOPS/PEDOT:PSS ratio of 11:1 (C4). This indicated that the reactive sulfonate groups in the PSS chains were fully bound by GOPS molecules, and the cross-linking reached a saturated status. Hence, a further increase in the GOPS amount did not lead to any additional cross-linking reactions. Consequently, the C4 composition was identified as the optimal one, being used in subsequent experiments.

**Figure 3:**
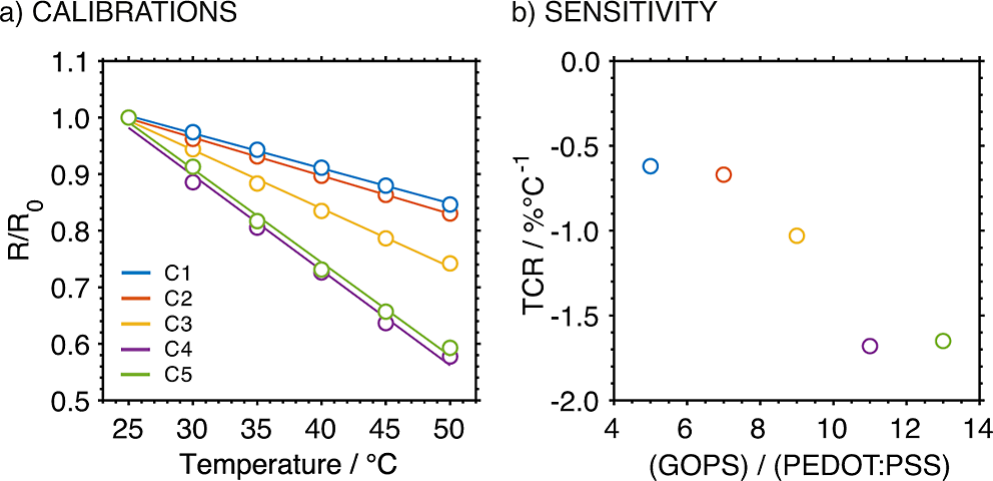
(a) Calibration curves observed for T-patches prepared using different weight ratios of GOPS to PEDOT:PSS in the preparation of the composite. The ratios for GOPS to PEDOT:PSS of 5:1, 7:1, 9:1, 11:1, and 13:1 is denoted as C1, C2, C3, C4, and C5, respectively. (b) Plot of TCR values obtained for different compositions.

### In Vitro Assessment of the Analytical Performance of the T-MN

Having accomplished the optimization of the PEDOT:PSS solution composition, the concept was translated into the MN format using the 3D-printed hollow MNs fabricated, as described in the Experimental Section. The result of the casting of the PEDOT:PSS solution inside the MN lumen was characterized with SEM images (Figure S3). The samples were prepared by cutting the MN from the tip going vertically, using a surgical blade, and then removing the cut tip from the substrate. A thin film of PEDOT:PSS (approximate thickness of 7 μm) was found to be deposited on the entire inner surface of the MN lumen. This is probably due to the cross-linking reaction that happens between the GOPS in the composite solution and the polyacrylate in the MN structure.

Additionally, the reproducibility of the PEDOT:PSS film was evaluated in terms of conductivity and thickness. The average resistance within different MNs was found to be 140.5 ± 12.8 kΩ (*n* = 5 T-MNs). Thus, the relative standard deviation (RSD) was calculated to be 9.1%. Being this value <10%, it can be concluded that an acceptable reproducibility was observed. Then, from the cross-section of the SEM images provided in Figure S3, the thickness of the PEDOT:PSS film was measured to be 7.13 ± 0.19 μm (*n* = 6 sensors, 10 positions in each of the MNs were measured and averaged). The variation within MNs was 2.7%, indicating the adequate reproducibility of the coating within MNs.

The evaluation of the analytical performance of the developed T-MN was conducted using the same setup as that described for the T-patch calibration (Figure S2). Additionally, to simulate the thermal diffusion from the ISF to the T-MN in on-body applications using the T-MN as a wearable, the in vitro assessment was performed by immersing only the needle tip part into the sample solution. In essence, the calibration was carried out by varying the temperature of the water bath using the controller of the commercial magnetic stirrer heater (IKA RCT basic). The multimeter (RS Pro, RS 985) was connected to the MN through the wires to measure the resistance.

Human body temperature varies within a narrow range depending on factors such as health status, gender, age, environmental temperature, and measurement sites. The normal body temperature is approximately 36.8 °C, while under pathologic conditions, it can drop below 36 °C (hypothermia) or rise above 38.3 °C (fever).^37^ Thus, the detection range of the developed T-MN was evaluated from 15 to 60 °C, not only to cover this range but also to expand it.

In this particular experiment, the *R*_0_ was reset to that obtained at 15 °C, and the *R*/*R*_0_ signal was calculated accordingly. The T-MN demonstrated good linearity, with an *R* ^2^ of 0.994 (Figure 4a, *n* = 3 MNs). The average TCR of the T-MN was determined to be −0.74 ± 0.05%° C^–1^, which is comparable to or even higher than other PEDOT:PSS-based temperature sensors reported previously in the literature (Table S2), but not in the MN format. These results revealed that T-MN can measure temperature variations under both healthy and pathologic conditions with high sensitivity.

**Figure 4:**
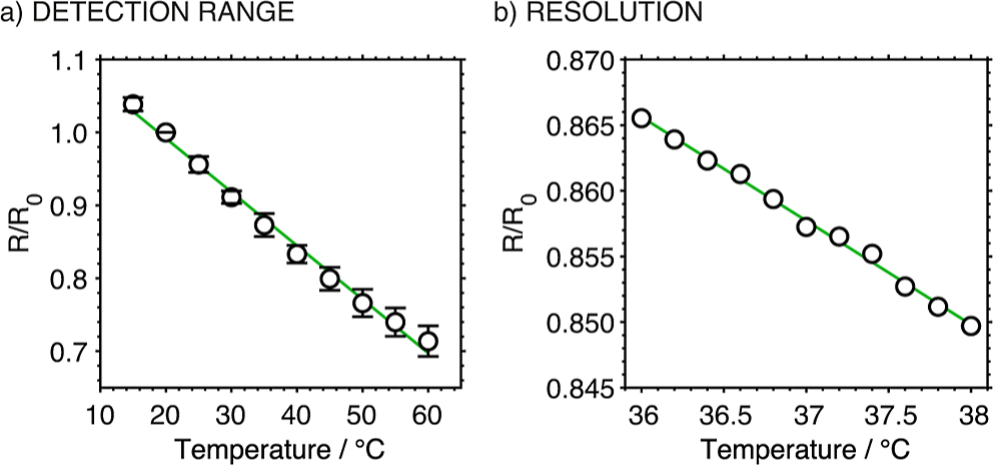
(a) Calibration curve for T-MN in the temperature range of 15 to 60 °C (*n* = 3, RSD = 6.7% and 0.7% for the slope and intercept, respectively). (b) Resolution study of the T-MN in a physiologically relevant temperature range, from 36 to 38 °C.

The resolution of the T-MN was investigated by gradually increasing the temperature of the water bath from 36 to 38 °C, measuring the resistance of the T-MN at every 0.1 or 0.2 °C increment. The calibration curve tested with a 0.2 °C increment (Figure 4b) presented a slightly better linearity than that with a 0.1 °C increment (Figure S3 in the Supporting Information): *R* ^2^ of 0.996 versus 0.991. It was observed that some of the data points deviated more from the linear regression curve with the 0.1 °C increment than for 0.2 °C. For example, the maximum deviation was found at 38 °C: 0.07% (corresponding to 0.1 °C) compared to 0.002% with the 0.2 °C increment. Consequently, it was demonstrated that the T-MN can accurately measure temperature changes with a resolution of 0.2 °C or even lower. This is indeed acceptable for real-world applications using the T-MN as a wearable sensor. For example, postsurgical continuous temperature monitoring to ensure that the recovery is progressing without signs of infection or complications requires such a resolution level.^38^

The repeatability of the developed T-MN was assessed by conducting three consecutive calibrations in the water bath from 20 to 40 °C, with 5 °C step increments and using a single T-MN. The average calibration is presented in Figure 5a. The RSDs for the slope and intercept were 2% and 0.3%, respectively, revealing good repeatability of the T-MN. The reproducibility was investigated by performing calibrations for five individual T-MNs from two different batches. The averaged calibration curve is depicted in Figure 5b. Notably, one of the batches was the same one shown in Figure 4a. Even changing the composite batch, the reproducibility was found to be rather good, with low RSDs for the calibration parameters (2% for slope and 0.4% for intercept).

**Figure 5:**
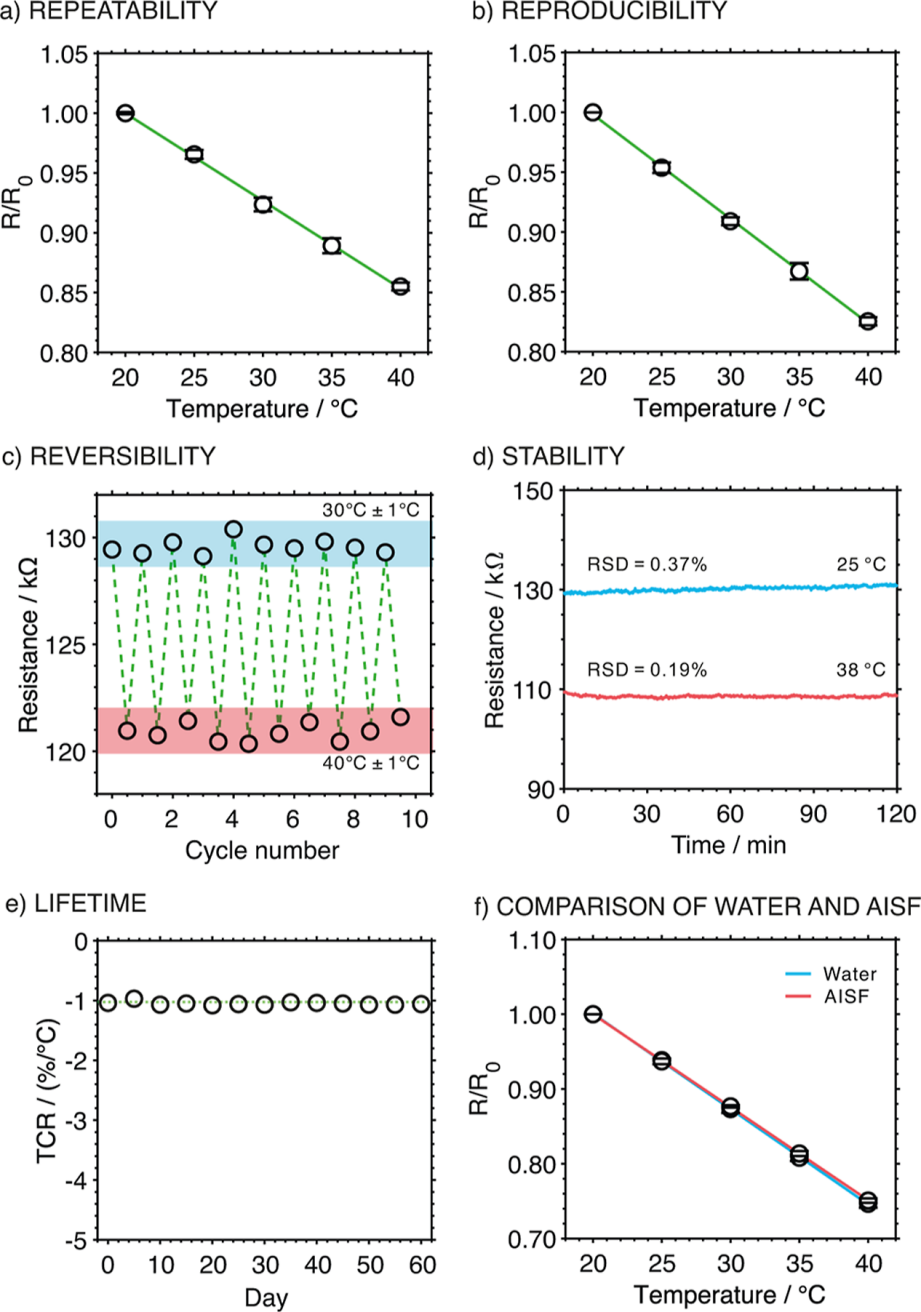
(a) Repeatability study performed with three consecutive calibrations using one T-MN. (b) Reproducibility study performed with two composite batches (*n* = 5 T-MNs). (c) Reversibility test carried out by varying the temperature from 30 to 40 °C for 10 cycles. The shaded regions represent the calculated range of resistance variation for the T-MN within the temperature fluctuation range of the water bath. (d) Medium-term stability at 25 and 38 °C in water. (e) Lifetime test in a 60 day period, accomplishing a calibration every 5 days. The TCR (sensitivity) was plotted versus the day of the calibration. (f) Calibrations of the T-MN performed in water and artificial interstitial fluid (AISF).

The reversibility of the T-MN was evaluated through measuring the resistance at varied temperatures from 30 to 40 °C for 10 consecutive cycles. Figure 5c displays the results. The RSDs for the measurements at 30 and 40 °C were found to be 0.3% and 0.4%, respectively, which correspond to a temperature variation of 0.41 and 0.50 °C. These outcomes manifested the good reversibility of the developed T-MN, even considering the rather drastic temperature changes in the experiment. Then, acknowledging the temperature fluctuations of the water bath (±1 °C according to the manufacturer) during the measurements, the found variations were likely attributed to this temperature change in the water bath rather than an intrinsic performance of the T-MN. Importantly, human body temperature fluctuates by 0.8–1 °C following circadian rhythms, peaking during the diurnal period and reaching its lowest point in the nocturnal time.^39^ Besides, body temperature may vary by 1–5 °C with physical exercises or changes in the environment.^40,41^ Consequently, a temperature sensor that can reversibly measure the temperature in these ranges without compromising its performance is highly convenient for on-body applications.

Regarding stability, Figure 5d presents the signals from continuous recordings at 25 and 38 °C. These temperatures were selected to mimic the lower and upper levels of the expected temperature range in the subsequent on-body measurements in euthanized rats. The T-MN exhibited acceptable stability during the 2 h continuous measurements, displaying RSDs of 0.4% and 0.2% at 25 and 38 °C, respectively, which imply temperature variations of 0.14 and 0.06 °C h^–1^. Notably, a longer stability experiment was not accessible with the utilized setup (temperature fluctuations are expected from set temperature due to the decay of the temperature control unit in the magnetic stirrer heater and water evaporation in the water bath). Overall, the results are suitable to ensure appropriate operational stability, which is crucial for temperature monitoring in real-world settings such as in intensive care units or during surgical procedures, where the sensor needs to operate with medium sampling rates (every several seconds to several minutes) for hours without experiencing significant drift or deviation in the readout signal that leads to significant errors.^42,43^

The lifetime of the developed T-MN was researched by performing calibrations (from 20 to 40 °C) every 5 days over a 60 day period. Figure 5e displays the plot of the TCR measured over such a period (the corresponding calibration curves are shown in Figure S5). T-MN exhibited negligible variation in the calibration parameters (RSDs of 3% and 0.6% for the slope and intercept, respectively). Consequently, the T-MNs can be used for at least 60 days without any sensitivity deterioration. It is worth noting that the T-MNs were stored in dry and dark conditions after each calibration to minimize any environmental influence on sensor performance. While the PEDOT:PSS is susceptible to degradation under thermal stimuli^44^ or light exposure,^45^ the plasticizing effect from GOPS^36^ or delamination of the composite film from the inner lumen surface of the hollow MN^46^ could also contribute to a potential change in T-MN performance over time. However, no significant performance changes were observed during the study period under the applied storage and testing conditions.

T-MN was conceived to monitor the temperature in the ISF, which has a complex composition. Thus, to understand any possible matrix effect on the response, the calibration was performed in artificial ISF (AISF), compared with the calibration conducted in water. As shown in Figure 5f, the calibration curves observed in both media overlapped, demonstrating nearly identical calibration parameters. This indicated that the heat transfer from the liquid medium to the T-MN is unaffected by the type of medium used. For ease of operation, the water bath was selected for experiments involving performance evaluation and analytical application.

### Skin Insertion and Biocompatibility Assessment

In its wearable format, the T-MN was conceived to penetrate the skin, which consists of several layers (including the outmost mechanically tough stratum corneum). The developed T-MN has a tip diameter <45 μm, a radius of tip curvature <50 μm, a tip angle of ca. 52°, a basal edge width of 1000 μm, and a needle length of 1000 μm (Figure 6a). In principle, these geometrical parameters ensure the appropriate penetration depth and painless insertion inside the skin.^47^

**Figure 6:**
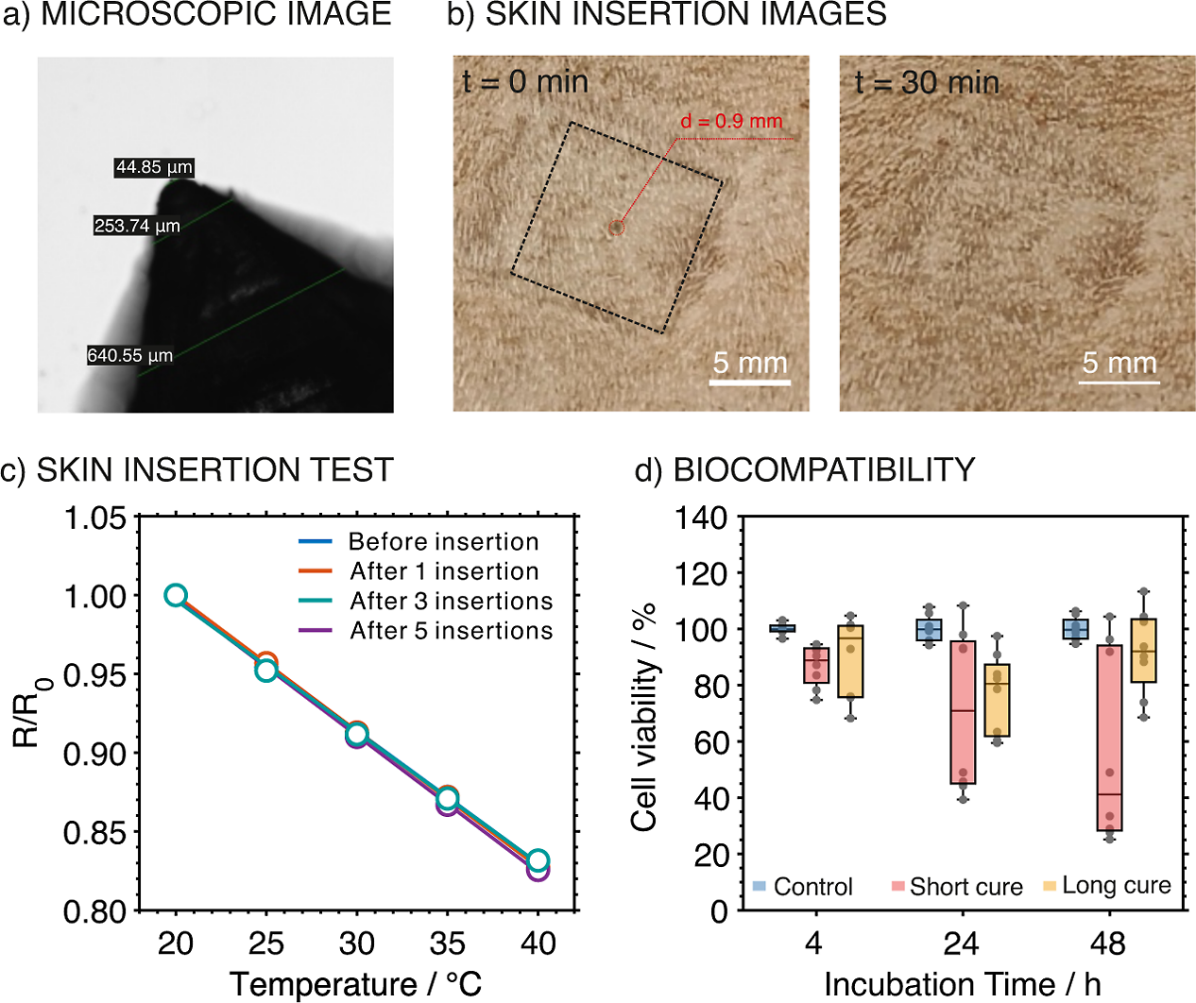
(a) Microscopic image of the tip part of the T-MN. (b) Images of the rat skin after T-MN insertion at *t* = 0 and *t* = 30 min. (c) Calibrations of the T-MN before skin insertion and after one, three, and five insertions. (d) Cytotoxicity test of the T-MN with different treatments over different durations (4, 24, and 48 h). Control groups were performed without adding the T-MN into the cell culture solution. “Short cure” implies that the MNs were prepared with a short period of UV curing (5 min) after printing, while “long cure” implies that the MNs were postprocessed with 30 min of UV followed by soaking in PBS overnight before adding into the cell culture solution.

Importantly, skin insertion with MNs is known to produce microchannels on the skin. It is expected that these microchannels can recover to their initial state soon after removing the MN to avoid any potential risk of infection.^48^ Accordingly, the invasiveness level of T-MN was evaluated using pieces of rat skin. Figure 6b shows the surface morphology of the skin obtained from a euthanized rat right after the insertion of the T-MN for ca. 1 min and after 30 min (i.e., *t* = 0 min and *t* = 30 min). Figure S6 displays the entire morphological evolution of the microchannel formed on the rat skin every 5 min. As observed, the microchannel gradually closed from 0 to 20 min (the hole diameter was found to change from 0.9 mm to be indistinguishable), this being attributed to the self-healing ability of skin and the minimal invasiveness of the T-MN. The microchannel completely disappeared after 25 min from the T-MN removal, indicating the full recovery of the skin. Considering that the rat skin is normally selected as the experimental model for humans due to its histological and physiological similarities, it could be concluded that the rapid recovery of the skin following the T-MN insertion can significantly enhance patient comfort and well-being, minimizing any infection risk.

Mechanical tests were performed for unmodified and PEDOT:PSS-modified 3D-printed MNs through axial direction compression with a strain rate of 0.5 mm/min. The force needed for the unmodified MN to be broken was 4.86 ± 0.12 N, while the modified MN exhibited a fracture force of 4.41 ± 0.71 N, both significantly exceeding the force required for skin penetration (0.1–3 N).^47^ The calculated stiffness values were 23.70 ± 0.47 kN/m for the unmodified MN and 22.81 ± 1.04 kN/m for the modified MN, further confirming the good mechanical properties of the fabricated MNs. The absence of a significant difference in mechanical properties between the modified and unmodified MNs suggested that the drop-casting process followed by oven drying to generate the PEDOT:PSS film has a negligible impact on the mechanical performance of the MN.

In addition, the performance of the T-MN after several skin insertions was evaluated by conducting calibrations before and after insertion into rat skin pieces. Figure 6c depicts the calibration curves before and after 1, 3, or 5 skin insertion(s). The calibration parameters showed negligible variations (RSD = 2% and 0.4% for the slope and intercept) after up to 5 insertions, suggesting the preserved integrity and unchanged sensing performance of the T-MN. Therefore, resiliency to skin penetration was confirmed. Moreover, the possibility for cytotoxicity effects while the T-MN is inside the skin was evaluated. Effectively, T-MN is expected to be safe for skin tissues, avoiding any possible toxicity to the surrounding cells after insertion. The experiment was accomplished using the 3-(4,5-dimethylthiazol-2-yl)-2,5-diphenyltetrazolium bromide (MTT) assay to quantitatively measure the cell viability after direct contact of the T-MN with human dermal fibroblasts.

Figure 6d presents the cell viability results for different experiment groups: the control group without adding the T-MN in the incubation solution as well as short cure and long cure groups corresponding to the MNs prepared by postprocessing with the short-term UV curing of 5 min or long-term UV curing of 30 min, this latter followed by soaking overnight in PBS. The cells were incubated for 4, 24, and 48 h in the presence of the T-MN. The cell viability for the short-term cured MNs showed a declining trend as the incubation time increased from 4 to 24 h and then 48 h. At the incubation time of 48 h, the cell viability was reduced to 57.1% ± 34.4%, suggesting that the short-term cured MNs are cytotoxic under long-term measurements of ≥48 h. Possibly this is due to the progressive leaching of uncured substances from the MNs substrate into the incubation solution.^49^ The large variation (SD = 29.1% and 34.4% for 24 and 48 h incubation, respectively) in the cell viability was presented in the short-sterm cured MNs, which is likely due to the excess of uncured resin in some of the MNs, caused by the inhomogeneous postcuring among different MNs.

The cell viability for long-term cured MNs followed by PBS washing presented no statistically significant differences (*p* values >0.05 using a paired *t*-test) for the tests at different incubation time spans, showing a mean viability of 90.0% ± 14.4% at 4 h, 77.0% ± 14.4 at 24 h, and 91.8% ± 15.2% at 48 h. Moreover, the 70% threshold was exceeded in all the cases, demonstrating the noncytotoxic nature of the developed T-MNs.^50^ From this set of experiments, it can be concluded that postprocessing, such as long curing time followed by soaking in PBS, can minimize the residues of the photoinitiator and uncured monomers that tend to remain in the 3D printed MNs. Indeed, short curing time has been demonstrated to lead to high cytotoxicity in previous studies.^51^ Notably, PBS washing is commonly used in the treatment of commercially available biocompatible resins.^52^ If necessary, there is room for future improvement using custom-made resins with noncytotoxic components serving as the substrate of the T-MN. Another possibility would be the direct printing of a composite-based substrate.

It is here hypothesized that once inserted in the skin, T-MN will provide a temperature value equivalent to the average of any infinitesimal temperature value read in each point of its surface since a gradient is expected inside the skin along its length. We simulated this situation with COMSOL, approximating the MN structure to either a 2D pyramid geometry or a 3D cone for comparison. The temperature gradient was calculated in these systems considering a variation of 2 °C from the surface until 1 mm in depth (i.e., the length of the MN). 2 °C has been reported as the maximum that can be found at such a depth from the skin surface.^18^ The temperature profiles are provided in Figure S7, accompanied by an explanation of the utilized COMSOL approach. From these results and considering typical temperatures of 34.3 °C for the skin surface and 36.3 °C at 1 mm of depth inside the skin,^53^ the average temperature that the MN will provide was calculated to be 34.63 and 34.36 °C for the 2D and 3D models, providing very similar values. Evidently, since the MN is bigger in its base than at the tip, the average temperature is closer to the value fixed for the skin surface.

### Influence from Fluctuations in the Environmental Temperature

During temperature measurements on humans using the T-MN wearable sensor, the resistance of T-MN must not be affected by fluctuations in the environmental temperature. Thus, a thermal insulating layer based on silicone foam was additionally applied to the upper part of the T-MN. The experimental setup used to investigate the environmental temperature influence on the response of T-MN is presented in Figure S8, allowing to change the externally and not at the MN tip. The results are shown in Figure 7a. Curve I presents the dynamic resistance changes of the T-MN with the insulating layer in the water bath, acting as the control experiment, and the calibration parameters were used to calculate the temperature variations in curve II, corresponding to the T-MN without the insulating layer. As observed in curve III for the T-MN with the insulating layer, the resistance was rather stable, and there was a negligible variation compared to the T-MN without the insulating layer (curve II). In this latter, the resistance exhibited a significant change as the environmental temperature varied (a variation of 2.6 °C as the environmental temperature changed from 23 to 30 °C and a variation of 5.9 °C from 30 to 40 °C during the measurement period).

**Figure 7:**
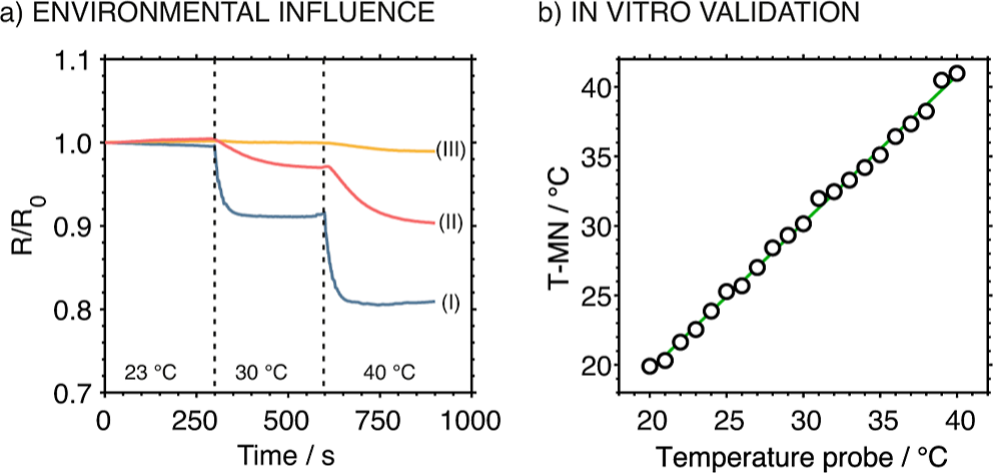
(a) Curve I: response of the T-MN with the insulating layer toward increasing temperature in the sample. Curve II: response of the T-MN without the insulating layer toward increasing environmental temperature. Curve III: response of the T-MN with the insulating layer toward increasing environmental temperature. (b) Correlation between temperature measurements from the T-MN and a commercial temperature probe (Pt100).

Before on-body temperature monitoring experiments were performed, the accuracy of the measurements provided by the T-MN was validated at the in vitro level. Figure 7b presents the correlation plot between the temperature measured by the T-MN and the commercial Pt100 temperature probe in an experiment accomplished by increasing the temperature of the water batch. A linear regression curve with a slope of 1.06, an intercept of −1.61, and Pearson’s coefficient of 0.999 was found, revealing a strong positive correlation.

### On-Body Temperature Monitoring in Euthanized Rats

On-body temperature monitoring using the developed T-MN was demonstrated in rats euthanized by CO_2_ inhalation. The rats were denotated by Karolinska Experimental Research and Imaging Centre (KERIC) from other research projects, i.e., not euthanized for our project. The results were validated with a commercial syringe-type optical temperature probe (Pyroscience, Germany), which was intradermally inserted into the skin and placed as close to the T-MN as possible (Figure 8a,b). The T-MN was placed on the abdominal region of the rat. All the T-MNs used in tests were calibrated before and after the experiments with 5-point calibrations. In all cases, no variations were found, suggesting the T-MNs were not damaged after skin insertion.

**Figure 8:**
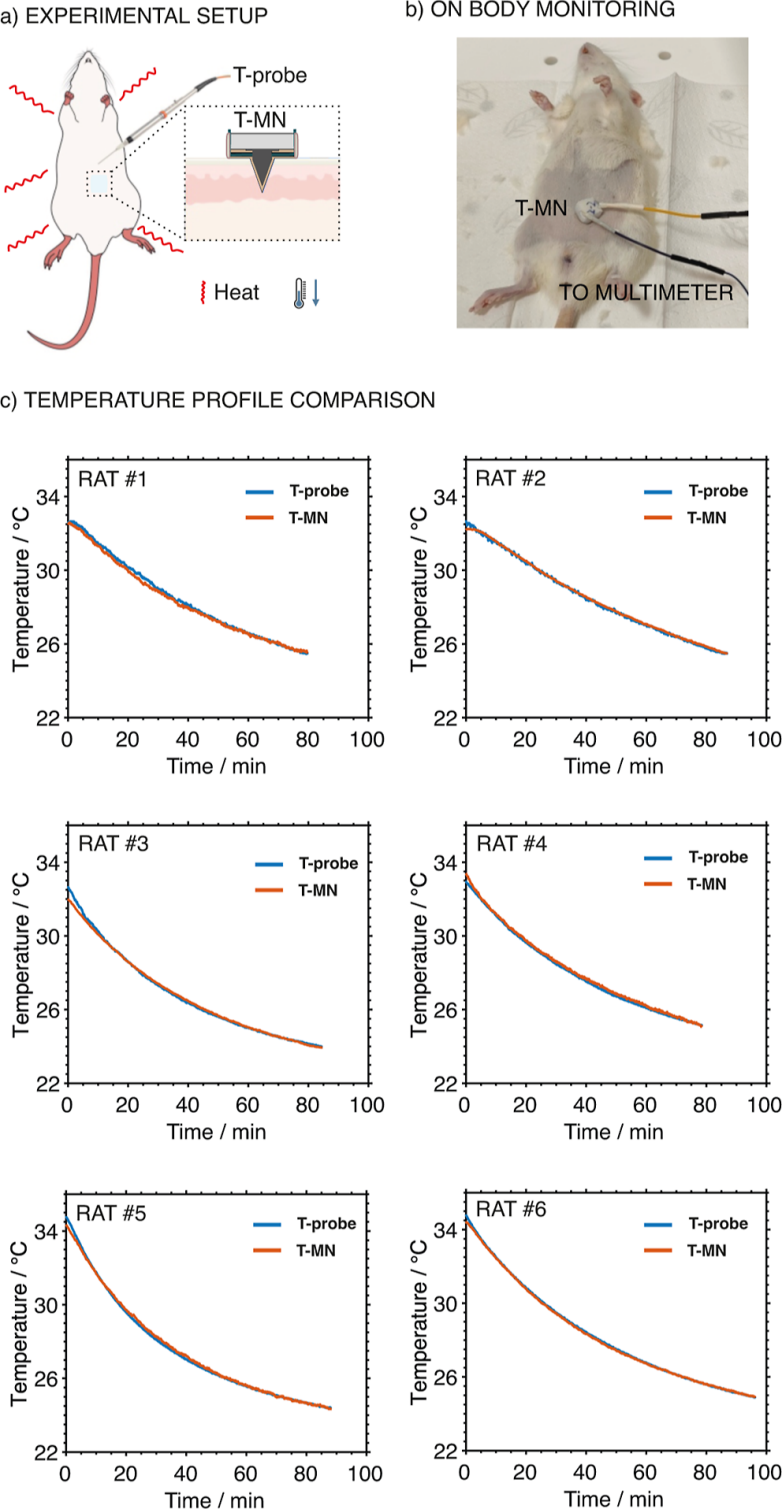
(a) Illustration of the on-body temperature monitoring in an euthanized rat using the developed T-MN and commercial syringe-type optical temperature probe (T-probe). (b) Real image of the temperature monitoring in a euthanized rat. (c) Temperature profiles obtained with the T-MN and the temperature probe in the six investigated rats.

It was expected that the temperature profiles obtained from the euthanized rats follow Newton’s law of cooling,^54^ as expressed by eq 2

2 $$T=\left(T_{0}-T_{env}\right)e^{-t/\tau }+T_{env}$$

where *T* is the temperature at time *t*, *T*_0_ is the temperature at the beginning of the measurement, *T*_env_ is the environmental temperature, and τ is the time constant of the system. As such, experimental data were fitted to this equation.

Figure 8c depicts the dynamic temperature profiles of 6 euthanized rats obtained with the T-MN and temperature probe, which was positioned at approximately 1 mm of depth from the skin surface (to coincide with the MN length). After euthanasia, the intradermal temperature of rats gradually decreased from 32–34 °C to a temperature close to room temperature (23 °C), owing to the shutdown of the thermoregulation system in the rat body. Notably, the initial recorded temperature is a bit lower than the normal abdominal temperature,^55^ which may be due to the temperature decrease during the sensor placement on the shaved abdominal region on rats after the euthanasia.

The nonlinear fitting of the obtained temperature profiles was conducted to quantitatively compare the accuracy of temperature monitoring using T-MN. The profiles obtained with the two different techniques revealed overlapped curves (Figure 8c) and rather similar fitting parameters (Table S3) in all the cases. Furthermore, discrete temperature values extracted from the continuous profiles at every 10 min interval for each rat showed only slight differences: average of −0.1–0.1 °C (Table S4). Overall, the results demonstrate a strong agreement between the temperature monitored by the T-MN and that recorded by the temperature probe, confirming the valid accuracy and reliability of the T-MN for intradermal temperature monitoring. Despite the temperature probe measuring at its tip region (positioned at 1 mm in depth) and the MN providing an average temperature of the touched ISF along its entire area (see above our hypothesis), this did not affect the observed profiles. Surely, the temperature gradient inside the skin within the first millimeters in depth is not significant enough to provide any differences between the probes.

## Conclusions

We introduced a 3D-printed microneedle temperature sensor (T-MN), showcasing a resilient and biocompatible architecture realized through high-resolution 3D printing and the integration of a PEDOT:PSS within the microneedle lumen. The performance of the sensor was comprehensively evaluated in vitro, demonstrating a broad detection range, exceptional repeatability, reproducibility, reversibility, and medium-term operating stability. Moreover, the T-MN exhibited sustained sensitivity for a minimum of two months. A thermal insulating layer was added to the rear of the T-MN to mitigate the impact of external temperature variations, resulting in exceptionally precise temperature measurements. These properties render the T-MN appropriate as a wearable platform, as evidenced by the validated monitoring of body temperature in a post-mortem animal model.

## Supplementary Material



## Notes

Experimental details; nonlinear fitting parameters of on-body temperature profiles; quantitative temperature profile comparison; experimental setup illustrations; and temperature patch preparation scheme (PDF)
