# Supplementary material for: Wearable 3D-Printed Microneedle Sensor for Intradermal Temperature Monitoring
Source: ACS Sens. 2025 Apr 15;10(6):4027–37. doi: 10.1021/acssensors.4c03681 (PMC12210253; doi:10.1021/acssensors.4c03681)
Supplement: Supplementary file 1 [file se4c03681_si_001.pdf]

Supporting Information for:

Wearable 3D-printed Microneedle Sensor for Intradermal Temperature Monitoring

Qikun Wei[1], Daniel Rojas[2], Qianyu Wang[1], Ruben Zapata-Pérez[3], Xing Xuan[2], Águeda Molinero-Fernández[2], Gastón A. Crespo[1,2] and María Cuartero[1,2,*]

[1]Department of Chemistry, KTH Royal Institute of Technology, Teknikringen 30, SE-114 28, Stockholm, Sweden.

[2]UCAM-SENS, Universidad Católica San Antonio de Murcia, UCAM HiTech, Avda. Andres Hernandez Ros 1, 30107, Murcia, Spain.

[3]Group of Metabolism and Genetic Regulation of Disease, Universidad Católica San Antonio de Murcia, UCAM HiTech, Avda. Andres Hernandez Ros 1, 30107, Murcia, Spain.

*Corresponding author: mariacb@kth.se

Table of Contents

Experimental section *3*

Reagents, materials, and instrumentation. 3

In vitro performance evaluation. 3

Cytotoxicity test 4

On body temperature monitoring on euthanized rats. 4

Tables *5*

Table S1. Sensitivity comparison 5

Table S2. Composition of the PEDOT:PSS solution 5

Table S3. Non-linear fitting parameters for temperature profiles 5

Table S4. Comparison of temperature values 6

Figures *8*

Figure S1. Schematic illustration of the planar T-patch preparation 8

Figure S2. Illustration of the experimental setup 8

Figure S3. SEM images of the cross section of the T-MN sensor 9

Figure S4. Calibration curve for resolution study 9

Figure S5. Lifetime test for T-MN. 10

Figure S6. Microneedle insertion on rat skin. 10

Figure S7. Simulation of temperature distribution along MN done in COMSOL 11

Figure S8. Illustrative setup for environmental temperature influence study. 11

References *12*

Experimental section

Reagents, materials, and instrumentation. Poly(3,4-ethylenedioxythiophene) polystyrene sulfonate (PEDOT:PSS, PH1000) was purchased from Ossila, Triton X-100 and (3-glycidyloxypropyl) trimethoxysilane (GOPS, laboratory grade) were purchased from Sigma-Aldrich (Sweden). All solutions were prepared in doubly deionized water (18.2MΩ cm^{-1}, Milli-Q water systems, Merck, Millipore) unless specially mention. Polyester film (0.100 mm in thickness) and conductive copper foil tape (Hi-bond 740, 0.07 mm in thickness) were purchased from RS Sweden. 3D printing resin (Clear Microfluidics Resin V7.0a) was obtained from CADworks3D microfluidics. Annealed stainless steel alloy wire (Fe/Cr18/Ni10, 0.075 mm) was obtained from GoodFellow. Super glue (Loctite Precision) was purchased from Clas Olhsen. Thermal insulating foam tape was purchased from RS Pro (Sweden). Double adhesive tape (3M 300LSE) was purchase from 3M Sweden.

PEDOT:PSS solutions were prepared by first mixing PEODT:PSS aqueous solution (1.3 wt%) with Triton X-100 (1.3 wt%) at the volume ratio of 2:1 and sonicated for 10 minutes, following by adding GOPS with corresponding weight ratio (PEODT:PSS : GOPS = 5:1, 7:1, 9:1, 11:1 or 13:1, which is noted as C1, C2, C3, C4 and C5, respectively) and then sonicated for 15 minutes to get a homogeneous solution. Artificial interstitial fluid (AISF, pH 7.4) was prepared as described elsewhere, with a composition of 20 g L^{-1} BSA, 3.5 mM KCl, 1.5 mM $CaCl_2$, 0.7 mM $MgCl_2$, 140 mM NaCl, 26 mM $NaHCO_3$, 1.7 mM Na_2HPO_4, 6 mM glucose and 7 mM urea.[1,2]

Geometrical parameters of T-MN were characterized by an optical microscope (Nikon Eclipse Ti2 inverted, Nikon Instrument, Inc.). Mechanical test of the microneedles was performed through axial direction compression using Instron 5944 universal testing machine (Norwood, MA, USA), utilizing the 10 kN load cell with the compression strain rate of 0.5 mm/min at room temperature. Apreo 2s field emission scanning electron microscope (Thermo Fisher Scientific, USA) was used to characterize the surface morphology of cast PEDOT:PSS film inside the microneedle lumen. The acceleration voltage and probe current were set to 1kV and 0.2 nA, respectively, while the working distance was kept as 11 mm.

In vitro performance evaluation. The calibration of T-patch was done in water bath by completely immersing the T-patch into water. The temperature of water was controlled using a magnetic heater while the resistance at different temperatures were measured by a multimeter. Calibration for T-MNs was performed in the similar way but with only the microneedle part immersed into water to better imitate the thermal diffusion scenario in real-world settings. For resolution study, the temperature of water bath was increased with a 0.1°C or 0.2°C increment interval from 36°C to 38°C and the resistance values at different temperatures was recorded by the multimeter. In repeatability and reproducibility test, the calibrations were conducted between 20°C to 40°C with the temperature interval of 5°C. Three consecutive calibrations were performed for the repeatability study and the resistance ratios was calculated by dividing the resistance at tested temperature by the resistance at 20°C (R_0). For reproducibility test, calibrations were done for five T-MNs from two batches. The reversibility test of T-MN was conducted by placing the T-MN in either of two containers filled

with water at different temperatures (30°C or 40°C) following the sequence of 30°C then 40°C and back to 30°C for ten cycles. The resistance of T-MN in each measurement was recorded after it reached to a stable value. Skin insertion test with calibration was conducted by performing calibrations in water bath before inserting the T-MN in rat skin sample and after 1, 3, or 5 skin insertions. Medium-term operational stability was tested by continuously measuring the resistance of T-MN immersing in water bath at different set temperatures (25°C or 38°C) for 2 hours. Lifetime test was carried out by calibrating the T-MN every five days in a 60-day timespan. Influence of environmental temperature fluctuation on the response of T-MN was investigated by recording the resistance of T-MNs with or without applying the thermal insulating tape under different mimicked environmental temperatures. The experimental setup is showed in **Figure S6**. In vitro validation of T-MN was performed by varying the temperature of water bath and measuring the corresponding resistance of T-MN. The temperature values were calculated using the calibration parameters from the calibration done prior the experiment and the validation was performed by a Pt100 temperature probe.

Cytotoxicity test. Cell viability test was performed with human dermal fibroblasts using the 3-(4,5-dimethylthiazol-2-yl)-2,5-diphenyltetrazolium bromide (MTT) assay. Cells were seed in 24-well plates at a density of 4×10^4 cells/well in Dulbecco's modified eagle medium (DMEM) supplemented with 10% fetal bovine serum (FBS) and penicillin / streptomycin. The cells were allowed to grow for 24 h and then exposed to 3D printed MNs, which are sterilized under a UV lamp for 30 minutes before adding into the cell incubation solution. Different incubation time durations, namely, 4h, 24h or 48h was applied. After incubation, cells were washed with PBS and then 500 µL of MTT solution (1 mg mL^{-1}) was added. After another 4 h of incubation to allow the conversion of MTT into formazan crystals, 500 µL of DMSO was added to solubilize the formed formazan crystals afterwards. The amount of formazan was determined by measuring the absorbance at 540 nm using a microplate reader.[3] Cell viability was determined by the amount of MTT converted into formazan crystal and quantified as a percentage compared to the control group.

On body temperature monitoring on euthanized rats. On body temperature monitoring experiments were performed at Karolinska University Hospital (Stockholm, Sweden) and with the assistance from the operation manager and personnel at Karolinska Experimental Research and Imaging Centre (KERIC). The rats were denotated by KERIC from other research projects, not specially euthanized for our project. The T-MNs were calibrated before on body measurement. The rat was euthanized in a plastic box connecting to the condensed CO_2. Then the fur on the abdominal region of rat was shaved to facilitate operations. T-MN was manually inserted into the skin. The syringe-type optical temperature probe was inserted into the skin and placed close to the T-MN. The room temperature was set to 23°C while the relative humidity was set to 30%. These environmental conditions were kept as constant during all measurements.

Tables

Table S1. Sensitivity comparison of common thermistors and PEDOT:PSS based temperature sensors.

Sensor composition	Sensor format	Sensitivity (% °C^{-1})	Reference
Pt	-	0.39	4
Copper	-	0.42	4
Nickel	-	0.67	4
PEDOT:PSS / PANI	Planar	-0.80	5
PEDOT:PSS / Graphene	Planar	-0.06	6
PEDOT:PSS / Silk sericin	Planar	-0.99	7
PEDOT:PSS on polyimide fiber	Fiber	-0.46	8
PEDOT:PSS on dying thread	Thread	-0.48	9
PEDOT:PSS on glass	Planar	-0.86	10
PEDOT:PSS / Triton X-100 / GOPS	Microneedle	-0.74	This work

Table S2. Composition of the PEDOT:PSS solution used for preparing temperature sensors.

Composition	1.3 wt% PEDOT:PSS / µL	GOPS / µL	1.3 wt% Triton X-100 / µL
C1	100	6.08	50
C2	100	8.51	50
C3	100	10.94	50
C4	100	13.37	50
C5	100	15.8	50

Table S3. Non-linear fitting parameters for temperature profiles obtained from temperature probe (labeled as T_0, T_{env} and τ) or T-MN (labeled as T_0', T_{env}' and τ') for the six investigated rats.

RAT #	T_0	T_0'	T_{env}	T_{env}'	τ	τ'
1	32.95	32.80	22.29	23.15	65.62	57.16
2	32.69	32.64	20.55	20.08	95.20	102.36
3	32.43	31.96	23.00	22.41	38.44	46.35
4	32.91	33.03	23.20	23.18	49.35	50.29
5	34.66	34.30	23.78	23.40	32.64	37.37
6	34.56	34.47	23.27	23.36	50.66	49.86

Table S4. Comparison of temperature values obtained by temperature probe (T-probe) or T-MN at different times over the investigated duration.

	Time / min	T (T probe) / °C	T (T-MN) / °C	ΔT / °C	AVE ΔT / °C
**RAT #1**	0.0	32.6	32.6	0.0	-0.1
	10.0	31.4	31.3	-0.1	
	20.0	30.1	30.0	-0.1	
	30.0	29.0	28.8	-0.2	
	40.0	28.1	27.9	-0.2	
	50.0	27.2	27.2	0.0	
	60.0	26.5	26.5	0.0	
	70.0	25.9	26.0	0.0	

	Time / min	T (T probe) / °C	T (T-MN) / °C	ΔT / °C	AVE ΔT / °C
**RAT #2**	0.0	32.7	32.2	-0.5	0.0
	10.0	31.6	31.6	0.0	
	20.0	30.3	30.5	0.2	
	30.0	29.4	29.4	0.0	
	40.0	28.4	28.6	0.2	
	50.0	27.7	27.8	0.1	
	60.0	27.0	27.1	0.1	
	70.0	26.4	26.4	0.0	
	80.0	25.8	25.9	0.1	

	Time / min	T (T probe) / °C	T (T-MN) / °C	ΔT / °C	AVE ΔT / °C
**RAT #3**	0	32.7	32.0	-0.7	-0.1
	10	30.2	30.1	-0.2	
	20	28.6	28.6	0.0	
	30	27.3	27.4	0.1	
	40	26.4	26.5	0.1	
	50	25.6	25.6	0.0	
	60	25.0	25.0	0.0	
	70	24.5	24.5	0.0	
	80	24.1	24.0	-0.1	

	Time / min	T (T probe) / °C	T (T-MN) / °C	ΔT / °C	AVE ΔT / °C
**RAT #4**	0.0	33.0	33.4	0.4	0.1
	10.0	31.1	31.2	0.1	
	20.0	29.7	29.7	0.1	
	30.0	28.5	28.6	0.2	
	40.0	27.5	27.6	0.1	
	50.0	26.7	26.9	0.1	
	60.0	26.1	26.2	0.1	
	70.0	25.6	25.6	0.1	

	Time / min	T (T probe) / °C	T (T-MN) / °C	ΔT / °C	AVE ΔT / °C
**RAT #5**	0	34.7	34.3	-0.4	0.0
	10	31.8	31.7	-0.1	
	20	29.6	29.7	0.1	
	30	28.1	28.2	0.1	
	40	27.0	27.2	0.2	
	50	26.2	26.3	0.1	
	60	25.6	25.6	0.0	
	70	25.1	25.0	-0.1	
	80	24.7	24.7	0.0	

	Time / min	T (T probe) / °C	T (T-MN) / °C	ΔT / °C	AVE ΔT / °C
**RAT #6**	0	34.8	34.4	-0.4	-0.1
	10	32.5	32.4	-0.1	
	20	30.8	30.8	0.0	
	30	29.5	29.5	0.0	
	40	28.4	28.3	-0.1	
	50	27.5	27.4	-0.1	
	60	26.8	26.7	-0.1	
	70	26.1	26.1	0.0	
	80	25.6	25.6	0.0	
	90	25.2	25.2	0.0	

Figures

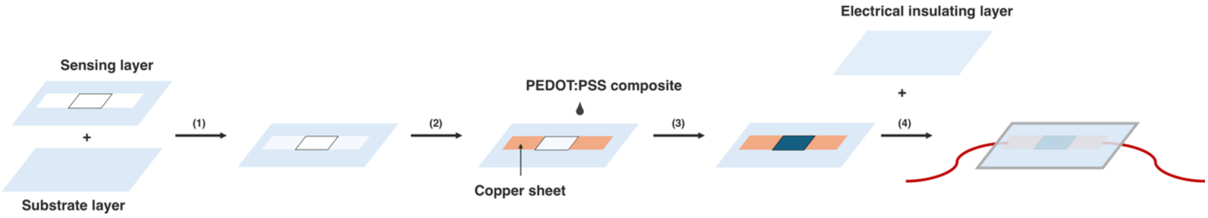

Figure S1. Schematic illustration of the planar T-patch preparation**:** (1) Assemble the substrate layer with the sensing layer using double-sided adhesive tape. (2) Copper sheets placement and PEDOT:PSS solution addition. (3) Oven drying at 100°C for 1 hour to obtain a homogeneous PEDOT:PSS film. (4) Connection wires constructing, apply the top electrical insulating layer, and seal the assembled patch with insulating hotmelt glue.

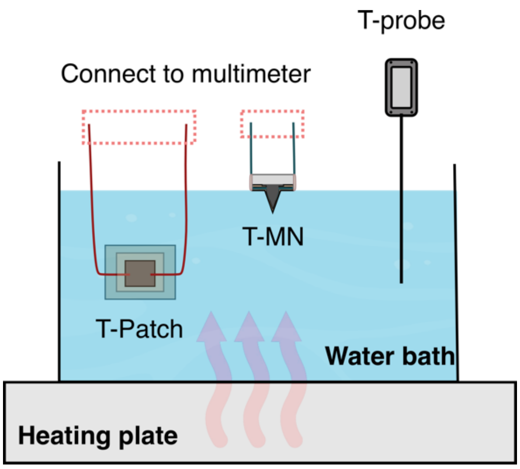

Figure S2. Illustration of the experimental setup for calibration of temperature sensors (T-patch or T-MN).

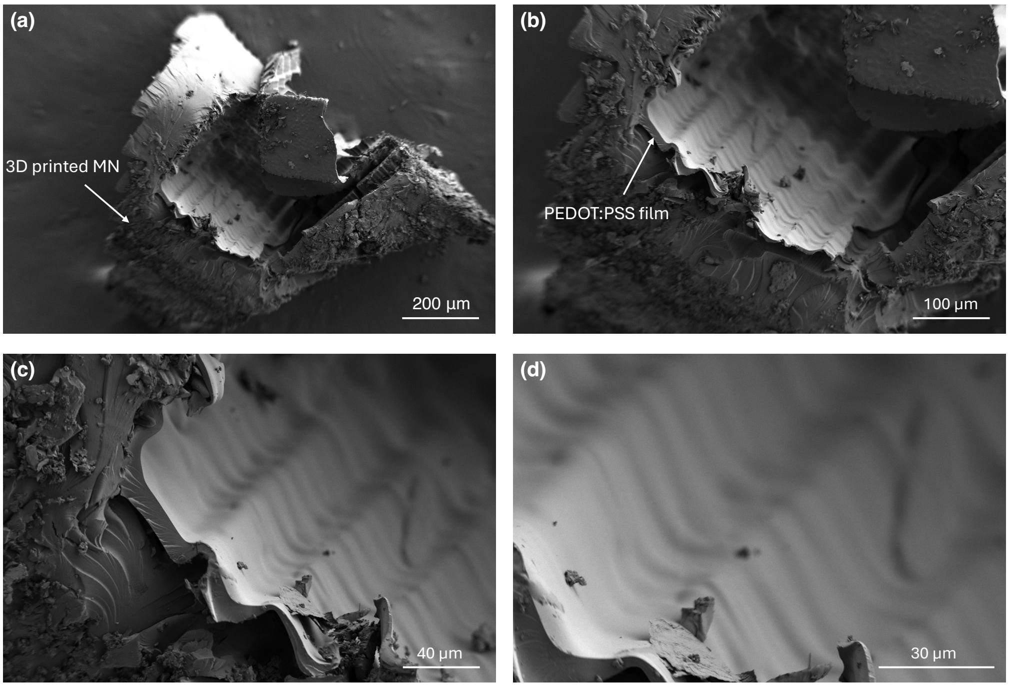

Figure S3. SEM images of the cross section of the T-MN sensor at different magnifications on the PEDOT:PSS surface: (a) ×100, (b) ×200, (c) ×500 and (d) ×1000.

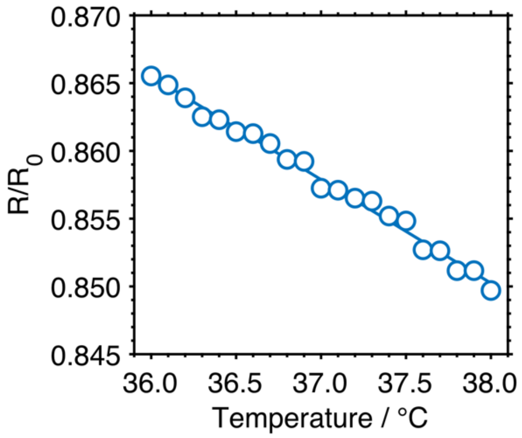

Figure S4. Calibration curve for resolution study of T-MN with 0.1 °C increment (R^2 = 0.991).

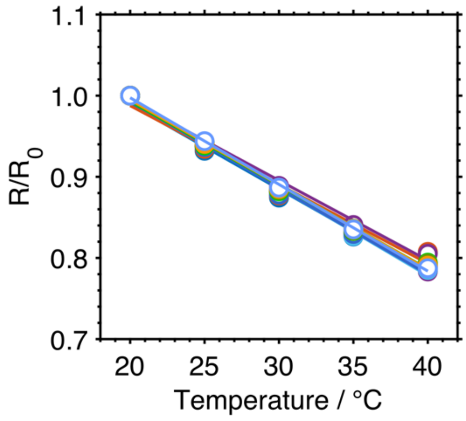

Figure S5. Lifetime test for T-MN. The calibration of T-MN was conducted every five days within a 60-day duration.

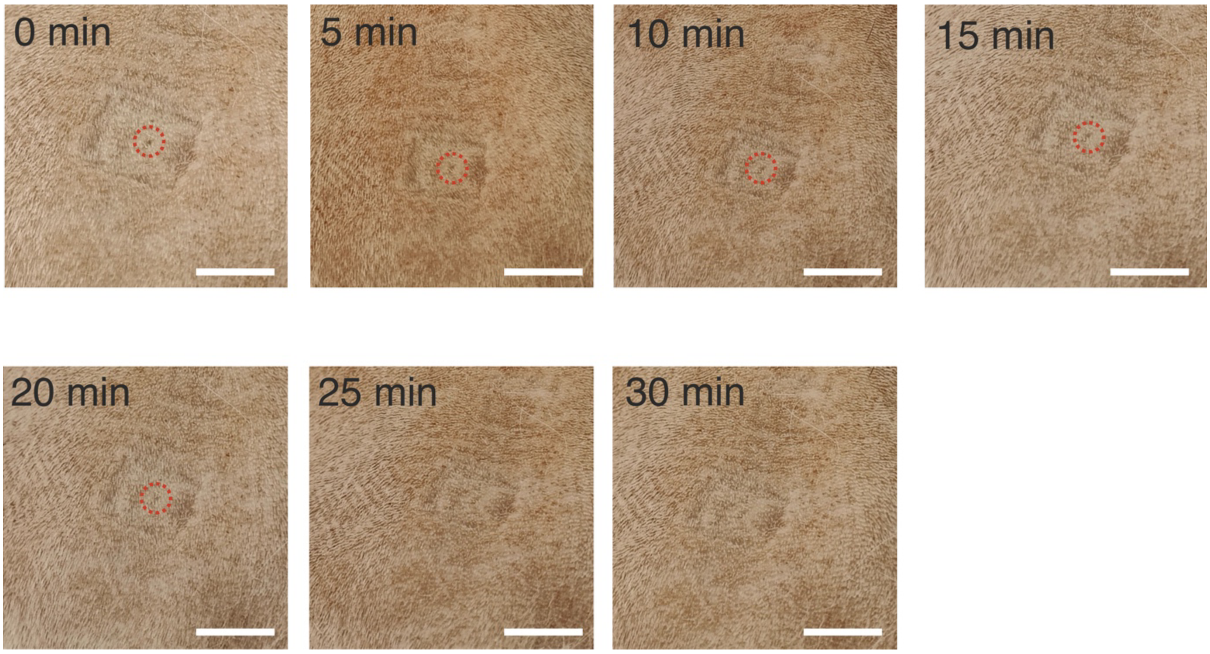

Figure S6. Microneedle insertion on rat skin. The morphology changes overtime of microchannel induced by T-MN insertion, scale bar: 1cm.

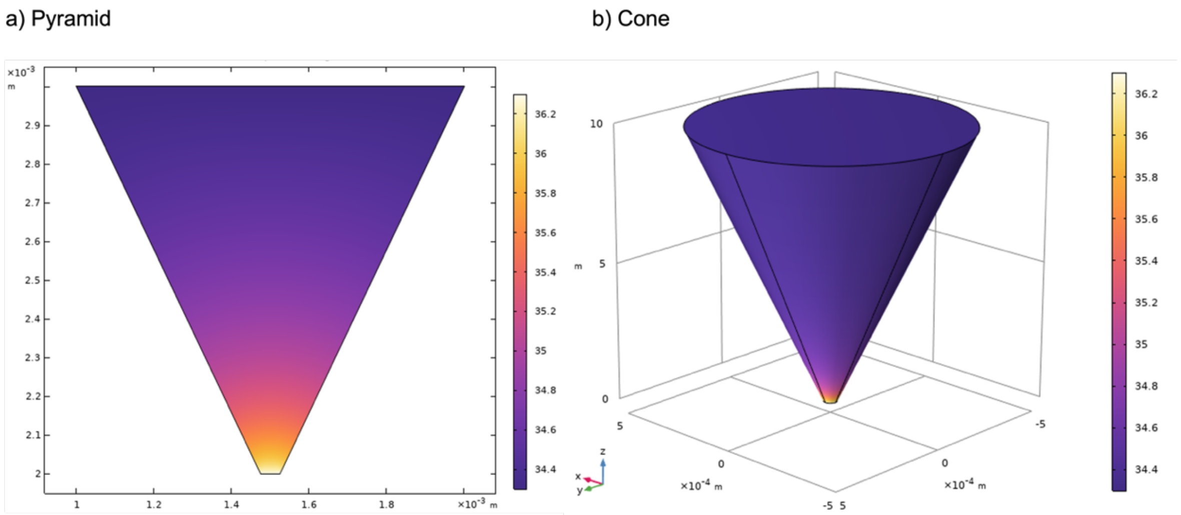

Figure S7. Simulation of temperature distribution along MN done in COMSOL. **a)** 2D plot representing the temperature distribution in a pyramid. Base: 1 mm. Tip: 0.05 mm. Height: 1 mm. A 2D model was built in COMSOL Multiphysics to simulate the temperature distribution in a pyramid geometry through the module Heat Transfer in Solids. **b)** 3D plot representing the temperature distribution in a cone. Base diameter: 1 mm. Tip diameter: 0.05 mm. Height: 1 mm. A 3D model was built in COMSOL Multiphysics to simulate the temperature distribution in a cone geometry through the module Heat Transfer in Solids. The simulations were performed considering a steady-state problem where the temperatures do not change with the time. It considers constant temperatures of 34.3°C for the base and 36.3°C for the tip. A thermal insulation is applied to the lateral segments of the pyramid: $-nq = 0$**.** Heat conduction in the solid (pyramid or cone geometry) was defined by the gradient temperature of $q = -k\nabla T$, where *k* is the thermal conductivity that was defined as 1 W/(m K). Notably, the change of this value did not influence the result in the temperature distribution. Then, *T* is the temperature in the base and tip. Specific parameters, such a heat transfer or density of the material, were not consider because we aimed for calculations assuming that each infinitesimal spot of the MN will exactly reflect the temperature in the skin position it is touching.

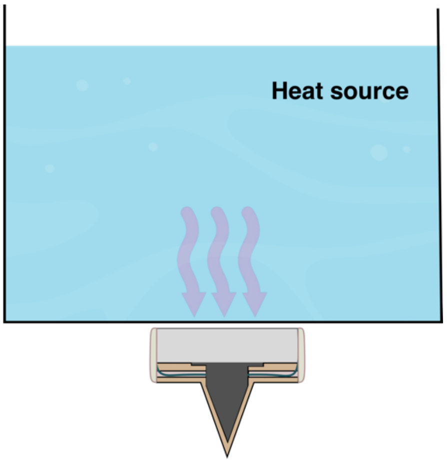

Figure S8. Illustrative setup for environmental temperature influence study.

References

(1) Fogh-Andersen, N.; Altura, B. M.; Altura, B. T.; Siggaard-Andersen, O. Composition of Interstitial Fluid. *Clin Chem* **1995**, *41* (10), 1522–1525.

(2) Bretag, A. H. Synthetic Interstitial Fluid for Isolated Mammalian Tissue. *Life Sci* **1969**, *8* (5), 319–329. https://doi.org/10.1016/0024-3205(69)90283-5.

(3) Kamiloglu, S.; Sari, G.; Ozdal, T.; Capanoglu, E. Guidelines for Cell Viability Assays. *Food Frontiers* **2020**, *1* (3), 332–349. https://doi.org/10.1002/fft2.44.

(4) *RTD elements and sensors - Introduction and tables*. https://www.omega.co.uk/temperature/z/resistanceelements.html (accessed 2025-02-20).

(5) Song, J.; Wei, Y.; Xu, M.; Gao, J.; Luo, L.; Wu, H.; Li, X.; Li, Y.; Wang, X. Highly Sensitive Flexible Temperature Sensor Made Using PEDOT:PSS/PANI. *ACS Appl. Polym. Mater.* **2022**, *4* (2), 766–772. https://doi.org/10.1021/acsapm.1c01224.

(6) Vuorinen, T.; Niittynen, J.; Kankkunen, T.; Kraft, T. M.; Mäntysalo, M. Inkjet-Printed Graphene/PEDOT:PSS Temperature Sensors on a Skin-Conformable Polyurethane Substrate. *Sci Rep* **2016**, *6* (1), 35289. https://doi.org/10.1038/srep35289.

(7) Pradhan, S.; Yadavalli, V. K. Photolithographically Printed Flexible Silk/PEDOT:PSS Temperature Sensors. *ACS Appl. Electron. Mater.* **2021**, *3* (1), 21–29. https://doi.org/10.1021/acsaelm.0c01017.

(8) Daoud, W. A.; Xin, J. H.; Szeto, Y. S. Polyethylenedioxythiophene Coatings for Humidity, Temperature and Strain Sensing Polyamide Fibers. *Sensors and Actuators B: Chemical* **2005**, *109* (2), 329–333. https://doi.org/10.1016/j.snb.2004.12.067.

(9) Lee, J.-W.; Han, D.-C.; Shin, H.-J.; Yeom, S.-H.; Ju, B.-K.; Lee, W. PEDOT:PSS-Based Temperature-Detection Thread for Wearable Devices. *Sensors* **2018**, *18* (9), 2996. https://doi.org/10.3390/s18092996.

(10) Nitani, M.; Nakayama, K.; Maeda, K.; Omori, M.; Uno, M. Organic Temperature Sensors Based on Conductive Polymers Patterned by a Selective-Wetting Method. *Organic Electronics* **2019**, *71*, 164–168. https://doi.org/10.1016/j.orgel.2019.05.006.
